# Association of masseter muscles thickness and facial morphology with facial expressions in children

**DOI:** 10.1002/cre2.431

**Published:** 2021-05-08

**Authors:** Christophe Guédat, Ourania Stergiopulos, Stavros Kiliaridis, Gregory S. Antonarakis

**Affiliations:** ^1^ Division of Orthodontics University Clinics of Dental Medicine Geneva Switzerland

**Keywords:** anthropometry, facial expression, facial muscles, masseter muscle, ultrasonography

## Abstract

**Objective:**

To evaluate the potential influence of muscular capacity and facial morphology on facial expressions in children.

**Materials and methods:**

A cross‐sectional study was carried out on 40 healthy children (ages 9–13), without previous orthodontic treatment. Masseter muscle thickness and anthropometric facial proportions were measured using ultrasound and digital calipers respectively. A three‐dimensional infrared face‐tracking system was used to register facial expressions. The maximal amplitude of smile and lip pucker (representing maximal lateral and medial commissure movement) were used for analysis. Stepwise regression was used to investigate whether muscle thickness or anthropometric facial proportions were associated with the quantity of commissure movement.

**Results:**

When performing maximal smile, children with thicker masseter muscles were found to have more limited displacement of the commissures (R = 0.39; *p* = 0.036). When performing lip pucker, children with thicker masseter muscles were found to have greater commissure movement (R = 0.40; *p* = 0.030). No significant associations were found between anthropometric facial proportions and facial expressions.

**Conclusion:**

Masseter muscle thickness seems to be associated with facial expressions in children. Those with thicker muscles show more limited commissure movement when smiling, but greater movement with lip pucker. This indicates that masticatory muscles may serve as a surrogate for mimic muscle activity. Facial morphology of the subjects does not seem to be associated with facial expression.

## 1 INTRODUCTION

The analysis of facial expressions in relation to orthodontic treatment has long been a question of interest in orthodontics (Reychler, 1965). Bibliographic analysis, however, shows that orthodontic research rarely focuses on such patient‐centered outcomes (Tsichlaki & O'Brien, 2014), despite the common understanding that research should measure the real potential benefit that is relevant to patients (Fleming et al., 2016). Facial expressions, which are integral to social interactions, may be of clinical importance to orthodontists and patients alike, and thus call for more research into this area.

With the advent of new technologies allowing three‐dimensional analyses, an evaluation of facial expressions based on two‐dimensional data can only provide limited utility. Some authors have furthermore suggested the necessity of the inclusion of the fourth dimension (i.e., the dynamic status over time) when evaluating the face [Trotman, 2011] or the smile (Sarver & Ackerman, 2003), which is very reasonable given the fact that in the context of social interactions dynamic stimuli may be interpreted differently from the static situation (Rymarczyk et al., 2016). The impact that dynamic facial expressions can have on our interactions with others are of great relevance.

Facial expressions, in relation to the different vectors of motion can be determined by the underlying hard tissues (craniofacial skeleton and dentoalveolar structures) (Trotman & Faraway, 2004), and the soft tissues (Uchida et al., 2018). The dynamic state of the soft tissues mainly depends on the recruitment of the muscles of facial expression, which contribute to the overall attractiveness of a smile (Lin et al., 2013). The resulting facial expressions follow a unique pattern of motion which seems to be consistent from childhood to young adulthood (Curti et al., 2019). When this motion is impaired, the resulting facial expressions may lead to psychological distress (Ishii et al., 2018).

The masseter muscle has been proposed to be a muscle that is representative of the masticatory muscles in general, based on computer tomography studies (Weijs & Hillen, 1984a; Weijs & Hillen, 1985). Moreover, masticatory muscles have been found to be associated with facial morphology, whereby those with a brachycephalic pattern have thicker muscles (Weijs & Hillen, 1984b). An indirect association has also been found with the activity of the masseter muscles having been shown to be associated with facial expressions, especially for smiling (Steele et al., 2018). In many individuals, the motor nerve to the masseter muscle has been shown to be activated during normal smile production (Schaverien et al., 2011). We can thus hypothesize that the activity of the neighboring muscles of facial expression may be related to the functional capacity of the masticatory muscles, and facial morphology, although this has never been adequately investigated. It would thus be interesting to identify individuals with a well‐developed facial musculature which may have an influence on orthodontic, surgical, and cosmetic treatment planning with regard to changes in soft tissue movement and facial expressions.

When a change occurs in the underlying hard tissues, either with orthopedic treatment (Antonarakis & Kiliaridis, 2019), or when combining orthodontics with orthognathic surgery (Al‐Hiyali et al., 2015; Johns et al., 1997; Nooreyazdan et al., 2004; Popat, Richmond, et al., 2012; Verze et al., 2011) the magnitude of facial expressions may be affected, making these facial expressions more similar to subjects without malocclusions of skeletal origin (Nafzinger, 1994).

Our hypothesis was that children with a well‐developed masticatory system, and a brachycephalic facial morphology, show greater perioral commissure movement when preforming facial expressions. This hypothesis is based on the aforementioned studies and the notion that the increased functional capacity of a muscle, or group of muscles, can lead to an increase in the range of motion. We therefore aimed to evaluate the potential influence of the masticatory muscular capacity as well as facial morphology on facial expressions in children.

## 2 METHODS

The present cross‐sectional prospective study was approved by our local research ethics board (No. 07–020), and all participants and their guardians gave informed consent.

### 2.1 Participants

The study sample consisted of 40 healthy children, without previous orthodontic treatment, seen at the University clinics of dental medicine in Geneva, Switzerland. Children were invited to participate in the study during their pre‐treatment diagnostic appointment, and for those who accepted and fulfilled the inclusion criteria, records were collected during a second appointment prior to commencing the orthodontic treatment. Eligibility criteria aimed to ensure the inclusion of subjects without striking deviations from facial norms. More specifically, inclusion criteria were the following: children aged between 9 and 13 years; mixed dentition; dental Class I or Class II malocclusion; and no previous orthodontic treatment. Exclusion criteria were the following: dental Class III malocclusion; transverse discrepancies; lip incompetence; non‐nutritive sucking habits; dysfunction or pathological signs of the temporomandibular joint; extreme brachycephalic or dolichocephalic facial pattern; craniofacial syndromes; neuromuscular disorders.

### 2.2 Methods

The three following variables were recorded during the same visit, by one investigator: masseter muscle thickness; anthropometric facial dimension; and facial expressions.

### 2.3 Masseter muscle thickness

Masseter muscle thickness was measured to the nearest 0.1 mm using an ultrasound scanner (Pie Medical Scanner 480, 7.5 MHz linear array transducer) adhering to the method described by Raasdsheer et al (Raadsheer et al., 1994). In brief, with the children seated and their heads in natural head position, the scan plane was perpendicular to the insertion of the masseter muscle, halfway between the gonial angle and the zygomatic arch. Two registrations of the transverse section of the muscle were taken on each side of the jaw, with the muscles in contraction (maximal clenching in intercuspidation). The average of the two measurements of the transverse section of the masseter muscle in contraction was used for the analysis.

### 2.4 Anthropometric facial dimensions

The anthropometric vertical facial proportions of the children were measured directly on the skin of the participants with digital calipers (FINO digital caliper P‐59112, FINO GmbH, Bad Bocklet, Germany) similar to what was proposed by a previous study (Raadsheer et al., 1996). The reference points on the skin were defined to approximate the underlying cephalometric landmarks of nasion, subnasale, and menton, thus allowing measurements for total facial height (distance from menton to nasion) and lower facial height (distance from menton to subnasale) to be measured. The lower facial height ratio was also calculated by dividing the lower facial height by the total facial height.

### 2.5 Facial expressions

Vectors of displacement of the oral commissures were recorded dynamically during a series of facial expressions with a three‐dimensional infrared face‐tracking system (Smarteye® Pro system, SmartEye AB, Gothenburg, Sweden) and the corresponding custom‐made MME (mimic muscle evaluation) add‐on software that allows the tracking of lip movements. The protocol and method used was that described in the paper of Sjögreen et al (Sjogreen et al., 2011). The method has been previously validated by another study (Schimmel et al., 2010).

Briefly, the participants were seated in a chair without a headrest to ensure a natural head position. A sequence of 10 photographs was taken for the identification of landmark settings. Subsequently, the reference position “rest position” was recorded with the lips at rest. Finally, two different facial expressions were recorded, each to its maximal extent, namely maximum smile and lip pucker. This task was repeated twice, in order to record the maximal commissure movements. The maximal amplitude of the smile (representing maximal lateral movement of the commissures) and of the lip pucker (representing maximal medial movement of the commissures) were used for the analysis.

Movements were looked at in the three axes, namely the x‐axis (horizontal commissure movement), y‐axis (vertical commissure movement), and z‐axis (anteroposterior commissure movement). Changes from rest position to maximal smile, or lip pucker, were recorded. Moreover, the resultant (R) was also calculated, which represents the combined three‐dimensional oral commissure displacement, considering the movement in all three axes. This was calculated with the following formula: R = √[(change in horizontal oral commissure movement)^2^ + (change in vertical oral commissure movement)^2^ + (change in antero‐posterior oral commissure movement)^2^].

### 2.6 Statistical analysis

All statistical analyses were performed using SPSS version 25.0 (SPSS Inc., Chicago, IL). Pearson correlation was used to analyze the correlation between the two facial expressions recorded, namely maximal smile and lip pucker, including age and sex as covariables. Similarly, Pearson correlation was used to analyze the correlation between masseter muscle thickness and anthropometric facial proportions. Stepwise regression was performed with soft tissue commissure movement as the dependent variable and the square of muscle thickness or anthropometric facial proportions as independent variables, along with age and sex. Masseter muscle thickness was squared for these analyses since the activity of this muscle depends on its surface area and not solely on its thickness. Soft tissue commissure movements were namely movements from rest to facial expressions (maximum smile or lip pucker).

## 3 RESULTS

**FIGURE 1:**
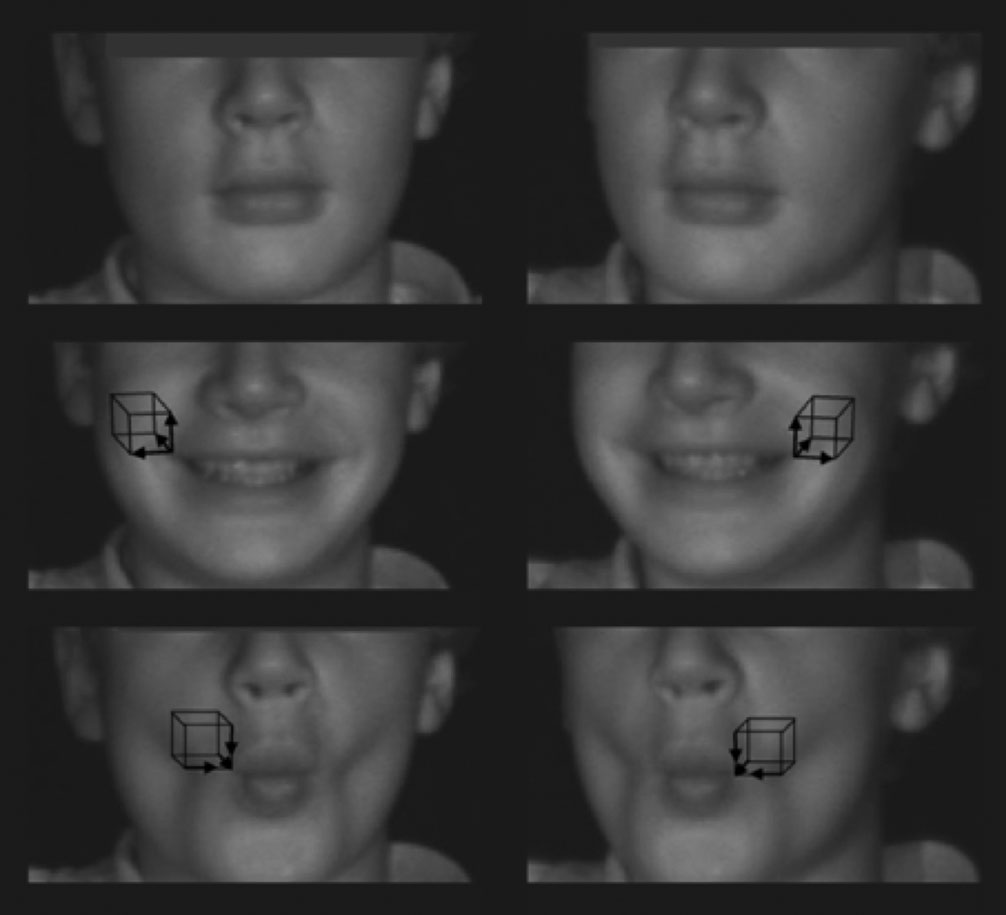
Direction of oral commissure movement in the x‐, y‐, and z‐axes from rest position (top image) to maximal smile (center image) and lip pucker (bottom image)

The participants consisted of 30 boys and 10 girls, with a mean age of 11.3 years (+/−1.7 years). All of the eligible participants accepted to take part in the study during the recruitment period, and an informed consent form was signed. Table 1 describes the participant demographics, anthropometric measurements and dynamic facial expression measurements of the cohort. Upon smiling (Figure 1), the oral commissures moved, on average, laterally (in the x‐axis), superiorly (in the y‐axis), and posteriorly (z‐axis). When performing lip pucker (Figure 1), the oral commissures moved, on average, medially (in the x‐axis), inferiorly (in the y‐axis), and anteriorly (z‐axis).

**TABLE 1:** Cohort descriptive data

			Mean	SD
Age (y)			11.3	1.7
Masseter muscle thickness (mm)			12.2	1.2
Anthropometric data	Total FH (mm)		124.3	24.7
	Inferior FH (mm)		43.2	8.7
	Superior FH (mm)		81.1	16.9
	Lower FH ratio (%)		34.8	2.7
Dynamic facial expression data	Rest mouth width (mm)		42.9	5.0
	Smile mouth width (mm)		58.4	4.8
	Pucker mouth width (mm)		28.4	4.7
	Smile	X‐axis (mm)	15.5	4.8
		Y‐axis (mm)	5.6	3.3
		Z‐axis (mm)	−12.9	6.6
		Resultant	20.9	6.3
		Relative mouth width change (%)	36.1	8.8
	Pucker	X‐axis (mm)	−14.5	4.9
		Y‐axis (mm)	−3.1	6.3
		Z‐axis (mm)	16.0	6.8
		Resultant	21.8	6.2
		Relative mouth width change (%)	−33.8	−9.4

Abbreviations: %, percentage; FH, facial height; mm, millimeter; SD, standard deviation; y, year.

An inverse correlation between maximal smile and lip pucker was found, with a significant Pearson correlation coefficient (−0.397; *p* = 0.037) when including age and sex as covariables. Children who showed greater commissure movement upon maximal smile showed less commissure movement when performing lip pucker and vice versa. No significant correlations were found between masseter muscle thickness and anthropometric facial proportions.

When performing multiple regression analyses using stepwise regression, looking at associations between masseter muscle thickness, anthropometric data, age, sex, and dynamic facial expressions, the following was found. No associations were found when looking at movement in a specific axis (x‐, y‐, or z‐axes) neither for maximal smile, nor for lip pucker. For the resultant (R) however, upon maximal smile, children with thicker masseter muscles had less displacement of the oral commissures (R = 0.39; *p* = 0.036) (Table 2), with none of the other independent variables showing significant associations. With regard to lip pucker, when calculating the R, children with thicker masseter muscles had greater oral commissure movement (R = 0.40; *p* = 0.030) (Table 2) again with no significant associations with any other independent variables. No significant associations were found between anthropometric facial measurements and facial expressions.

**TABLE 2:** Stepwise multiple regression analyses, with the resultant of oral commissure movement during facial expressions as the dependent variable, and masseter muscle thickness as the only significant independent variable

	R	*p*‐value	Beta coefficient	Constant
Resultant (maximal smile)	0.385	0.036*	−0.076	28.502
Resultant (lip pucker)	0.397	0.030*	0.077	−7.972

*Note*: Excluded variables from stepwise multiple regression: age; sex; lower facial height ratio.

^*^ Statistically significant (*p* < 0.05).

## 4 DISCUSSION

The present study evaluated the association between masseter muscle thickness, anthropometric facial measurements, and facial expressions (namely maximal smile and lip pucker) in children. Correlations were found between the three‐dimensional movement of the oral commissures and masseter muscle thickness both for lip pucker and maximal smile, however not in the same direction. Thicker masseter muscles, in children, are associated with greater oral commissure movement during lip pucker, but less movement when smiling. In addition, children with more oral commissure movement during smile displayed less movement during lip pucker and vice versa.

Our findings partially confirmed our hypothesis, that children with a well‐developed masticatory system, and a brachycephalic facial morphology, show greater perioral commissure movement when preforming facial expressions. This was only the case with regard to the lip pucker, but not for maximal smile. Anthropometric facial proportions, in the present sample, did not show associations with facial expressions. Facial expressions were also not associated with age or sex.

Interestingly, no associations were found between masseter muscle thickness and facial expressions within this sample when subdividing oral commissure movements into their individual components in the horizontal, vertical, and antero‐posterior axes. Only when looking at the total three‐dimensional commissure movement (which includes movement in the three planes of space) were significant associations observed. Perhaps the differences were too small to be significant in each individual plane, and combining them all together as suggested by Sjögreen et al (Sjogreen et al., 2011). permitted associations to be detected.

To the best of our knowledge, these data are the first which attempt to investigate whether the masticatory muscles are associated with soft tissues activity when performing facial expressions. Even though the muscles of mastication do not typically belong to the muscles of facial expression, we were interested in seeing whether they could perhaps be used as an indirect marker of the state of the muscles of facial expressions, since the masseter muscle is easily and reproducibly evaluated using ultrasonographic muscle thickness measurements. If the masticatory musculature is well developed, then by extrapolation perhaps the muscles of facial expression will also be well developed. The conflicting results between the two different facial expressions looked at however, puts the plausibility of such a hypothesis in doubt, since children with thicker masseter muscles showed greater soft tissue movement during lip pucker but less during maximal smile than those with thinner muscles.

When looking more specifically into the activity of the muscles of facial expression recruited when performing each of these facial expressions, it can be seen that different muscles are brought into play. The act of lip puckering mainly depends on the activation of the orbicularis oris muscle (Jain & Rathee, 2020). This muscle originates on each side from the modiolus for its deeper part and from the other muscles of facial expression for its superficial part (Nicolau, 1983).

Smiling, on the other hand, implies the recruitment of the elevation muscles of the commissures which coalesce at the modiolus. They are mainly controlled by zygomaticus major and the levator anguli oris muscles, pulling them in a superolateral and posterior direction for the zygomaticus major and with an additional superior vector for the levator anguli oris, increasing the elevation (Dao & Le, 2020; Ewart et al., 2005). Because the prominence of the zygomaticus major is more important than the levator anguli oris muscle, a well‐developed musculature of both muscles can diminish the vertical amplitude of the movement due to the levator anguli oris muscle. This could partially explain our results, with regard to less oral commissure movement upon smiling in children with thicker masseter muscles. This limited upward pulling effect of the commissure when smiling in the presence of a well‐developed musculature was also observed by Kant et al (Kant et al., 2014). A negative correlation was found between the thickness and the activity of the orbicularis muscle (whose contraction occurs when smiling) in healthy individuals. Moreover, a direct link between the masseter and the modiulus muscles does not exist, also supporting our findings. Well‐developed muscles of facial expression may limit the upward pulling effect of the smile and increase the movement when performing lip pucker.

A direct connection between the masseter and modiulus muscles may occur via the risorius muscle, but this is found in only two thirds of the population and is often described as a thin and wispy muscle (Som et al., 2012). This may not be important in relation to our results. However, the superficial musculoaponeurotic system, is another possible direct link between the two muscle groups, and this connective tissue in the oral region pulls the skin in the direction of the masticatory muscles, especially the buccinator muscle (Hinganu et al., 2018), which supports our results by limiting the upward pulling effect when smiling in the presence of thick masseter muscles.

In our sample, no association was found between the anthropometric data and oral commissure movement during facial expressions. Trotman et al. found that the general pattern of the movement of the face follows the static facial shape, except when performing lip pucker (Trotman & Faraway, 2004). Based on these findings, an association was expected between anthropometric facial measurements and smiling movements, but none was found. In line with our results however, Ramires et al. note that anthropometry based on vertical facial type determination is not a good predictor (Ramires et al., 2011), which can lead to the impossibility of showing any association between facial expressions and anthropometric facial measurements in our study.

With regard to age, Parks et al. observed a reduction of the strength and endurance of the orbicularis oris muscle with aging (Park et al., 2018). This could affect facial expressions, especially lip puckering. Houstis et al. compared facial expressions in children and in adults finding differences between the two groups (Houstis & Kiliaridis, 2009). Our results however found no association between facial expressions and age, perhaps because of the relatively homogenous age of the sample. Differences between males and females when performing facial expressions have been found for adults, but not for children (Houstis & Kiliaridis, 2009). Our sample, consisting only of growing children, also showed no differences between males and females.

Limitations of the present study generally related to generalizability. Included participants were all patients presenting to our orthodontic clinic and do not necessarily constitute a representative sample of the population. Moreover, only patients with non‐extreme facial patterns were included. Geographical and ethnic differences may exist, as well as differences in different malocclusion groups. With regard to the dynamic oral commissure data, the generation of facial expressions in a research environment is often artificial and difficult to reproduce, and this is especially true for the smile and less so for the lip pucker (Johnston et al., 2003). Lastly, we did not have cephalometric radiographs available to assess hard tissues morphology for the obvious reason of unnecessary radiation exposure. However, when cephalometric radiographs are not available to evaluate the hard tissues, anthropometric proportions can be used as an alternative, albeit with certain limitations (Budai et al., 2003; Farkas et al., 1999). Farkas et al. showed that errors in landmark positions are more frequent for vertical anthropometric data compared to cephalometric data, and thus these results should be interpreted with caution (Farkas et al., 1999).

The lips play a critical role in facial expressions (Piccinin & Zito, 2020). Most studies looking at facial expressions report on how medical intervention can normalize an affected pattern of motion, in pathological situations such as facial palsy (De Stefani et al., 2019). Looking at normal populations and the variation that exists in facial expressions however between individuals, and with aging, is essential to understanding the effect of disease on facial expressions and the potential benefits of the correction of these problems with techniques such as facial reanimation surgery. Common facial expressions such as the smile are important to analyze, but reproducibility is also key. Popat and colleagues have raised this problem and suggest the use of the words “puppy” and “baby” as the most appropriate gestures to register lip movement (Popat, Richmond, et al., 2008; Popat, Henley, et al., 2010). Moreover, facial landmark placement, in the x‐, y‐ and z‐coordinate system must be precise, and a reproducibility of <1 mm is considered clinically acceptable (Toma et al., 2009). These are important points to consider for future investigations.

The present results are pertinent to patients and practitioners alike. As patients are becoming more actively involved in treatment‐planning decisions, a more dynamic and individualized approach to analyzing their smile and facial expressions may provide for more interactive patient‐practitioner joint treatment planning, in fields such as orthodontics, orthognathic and plastic surgery, and cosmetic interventions. Care providers should be aware of different facial expression trends based on muscular factors, especially with regard to smile variation.

## 5 CONCLUSIONS

1. Thicker masseter muscles in children are associated with greater commissure movement during lip pucker.
2. Thinner masseter muscles in children are associated with greater commissure movement during maximal smile.
3. In addition, children with more commissure movement during smile display less commissure movement during lip pucker and vice versa.
4. Masticatory muscles seem to be associated with the activity of the muscles of facial expressions and may serve as a surrogate for the activity of these muscles.
5. Neither facial morphology, nor age nor sex are associated with facial expression in the present sample.

## CONFLICT OF INTEREST

The authors declare that they have no conflict of interest.

## AUTHOR CONTRIBUTIONS

Stavros Kiliaridis and Gregory S. Antonarakis were involvedin the conception, design and supervision of the study. Christophe Guédat, Ourania Stergiopulos, and Gregory S. Antonarakis were involved in data acquisition, analysis and interpretation. All authors contributed to the drafting of the manuscript and its critical revision, and gave final approval of the version tobe published. All authors are accountable for all aspects of the present work.

## Data Availability

The data that support the findings of this study are available from the corresponding author upon reasonable request.
